# Mr. Atkinson, on Apoplexy

**Published:** 1802-05

**Authors:** James Atkinson


### To the Editors of the Medical and Phyfical Journal.

Gentlemen,

I Now return to anfwer fome particulars of Dr. Langlow's paper, (Vol. vii. p. 281.) Univerfity honors, even when *regularly* and *fairly* obtained, certainly do not fanction intemperate language in the poffeffor, no more than riches the perpetration of crimes. The Doctor muft be fenfible that thofe obfervations at the conclufion of my firft communication on Apoplexy, cannot reafonably be applied to every gentleman in poffeffion of a DEGREE! I have the higheft veneration for many, whofe talents and genius give luftre to the profeffion; but I entertain an abhorrence of illiberal farcafm, and unmanly reflections. A DIPLOMA has not the powers of the Ægis, defcribed by the poets; nor has any one man the exclufive right of employing the fhafts of ridicule. Thofe who in controverfy maintain an independence of reafoning, and who fupport the important character of Phyfician, or Surgeon, with feeling and humanity, muſt ever meet the approbation and eſteem of all good men. Mr. Crowfoot's conduct in refuſing an interview might give riſe to the ſeverity of Dr. Langſlow. Theſe are points which have been ſufficiently handled, and have met with ſufficient, perhaps unmerited, cenſure.

Morgagni ſomewhere ſtates that one cauſe of Apoplexy may be, air eſcaping from the blood, and preventing the free circulation in the ſmall arteries of the brain! Pyrrho (Vol. vii. p. 336) ſuggeſts that Apoplexy may originate in the lungs! Oher Phyſicians, he ſays, imagined the toes might be affected ſo as to produce a kind of Apoplexy! But if one was fond of theorizing, the following equally plauſible hypotheſis might not be totally diſregarded. That Apoplexy depends on a torpor of the heart and arteries, or an idiopathic affection of the valves of the veins; which from ſchirroſity, ſpaſm, and many concurring cauſes, may ſuffer a temporary retrograde motion of the blood! In this manner might be accounted for all ſuddenly fatal diſeaſes, where the patient falls precipitately to the ground. In common caſes of ſyncope every power of action in the heart and arteries ſeems extinguiſhed, and when the patient begins to ſhew ſigns of life, the pulſations of the heart are irregular and fluttering, as if ſtruggling to recover ſome oppreſſive interruption of its energy. From defect of ſtimulus in the medullary ſubſtance, will be occaſioned acute throbbing, and a ſenſation of ſtricture in the head; and the reſpiration, from ill oxygenated blood in the lungs, will be labourious and difficult. But "imagination is a buſy power," and we bend our theories to our practice, as a mechanic would bend twigs for the uniformity of a machine!

They ſay our notions change with our philoſophy. Amongſt the ancients, other ſciences made more rapid ſtrides to improvement than that of medicine. The mathematical philoſophers and phyſicians ſtudied much of mechanics, and they thought themſelves wonderfully ingenious, when they began to explain the living body on mechanical principles. In proſecuting their enquiries on fermentation, glands, and humours, and of the heart pumping blood, their practice was uniform though their philoſophical terms appeared vague and obſcure. We are not, even in this age of refinement, free from ſimilar errors. I ſhall endeavour to illuſtrate theſe ſentiments from a caſe related by Dr. Fothergill, as there is nothing in mechanics* which can agree with the functions of organic life.

"A gentleman was ſeized with an apoplectic fit, one day as

* Haller, ſpeaks of the human body as an hydraulic machine.

as he was croffing the Thames in an open boat; the waterman landed him as quick as poffible, at the place he was going to, where all poffible affiftance was procured expeditioufly, and he foon recovered." The Doctor fays, "I faw him at his own houfe, foon after his recovery, and inquired if he recollected the pofture he was in when he loft himfelf: He replied, he was looking at a fhip, and kept his eye upon her after he had gone by her, till he loft himfelf, and fank down in the boat." On the perufal of this ftatement there is one queftion to afk, Was this *really* Apoplexy or not? A perfon unufed to the water, in paffing by a fhip, and looking ftedfaftly at the rigging, will, in any attitude, fometimes have vertigo and vomiting, even to fuch a degree that he will fink down, and lofe all recollection of his fituation. Dr. Fothergill was not prefent during the feizure, and it is poffible he might not be accurately informed of the fymptoms. However, he concludes, that in fhort-necked people looking backwards any length of time favours apoplexy, as in the above cafe, and explains his ideas to the following purpofe. He compares the carotid arteries and jugular veins to hollow flexible tubes of leather, or any other yielding fubftance, and the fhorter the tubes the more liable they are on being turned round of having their cavities compreffed. "The carotid arteries lying nearer the centre of motion are very little affected by the turn of the head, even in very fhort necked people; they continue to convey full ftreams of blood to the head: But the jugular veins lie nearer the furface, and if the neck is fhort, and full at the fame time, the twift fo far contracts their diameters that it is impoffible for them to return a proportionable quantity." The confequence is giddinefs and perfect Apoplexy. This is a circumftantial detail of a theory which Dr. Fothergill, no doubt, conceived to be juft. It is well known he was a man of erudition, and of liberal views; his opinions have the greateft weight with many refpectable practitioners, and his works have acquired the celebrity they fo juftly deferved; I reverence, with numbers, the excellency of his character, and fubmit the annexed Comments on a production of his, with diffidence, to the candid and impartial.

Every one muft allow that his doctrines of the leathern tube, and compreffion of the aorta, are very mechanical, and therefore inapplicable to the living fyftem. Where is the confent and fympathy of parts, the inherent contractile and mufcular power in any inanimate unorganized material, which can admit of any analogy with what the worthy Doctor has premifed? He advifes fhort-necked people always to turn their whole bodies towards the object they wifh to view, and not look over their their ſhoulders, as this attitude occaſions an obſtruction in jugular veins! Did the carotid arteries or jugular veins bear any compariſon to the flexible leather tube which he deſcribes, I ſhould be ſurpriſed that caſes of Apoplexy ſhould be ſo unfrequent.

Dr. Fothergill's Eſſay was profeſſedly written to diſcountenance the operation of bleeding in Apoplexies, and many of his reaſons againſt that practice are the reſult of his own experience. I think alſo profuſe general bleeding peculiarly hurtful to the conſtitution, and very probably may induce an incurable hemiplegia; but topical, never. A ſmall quantity abſtracted from the arm cannot be prejudicial, yet may be of eſſential ſervice: But I muſt deny that the diſeaſe can be produced by a large indigeſted meal! If the patient in that ſtate even falls down on the floor, it is not Apoplexy, and emetics may then be judiciouſly adminiſtered. And I believe, when the ſtomach is diſtended with food, it commonly projects forwards, and in pregnant women is puſhed upwards againſt the diaphragm; ſo that the aorta deſcendens cannot ſuffer by its compreſſion. The aorta only emerges into the abdomen from between the crura of the tranſverſe ſeptum, which is behind the ſeat of the ſtomach and above the crura, it is ſufficiently defended by the diaphragm. Mr. Crowfoot, from the authority of Dr. Fothergill and others, doubted not the propriety of giving emetics in Apoplexy,* as he perhaps never cloſely inveſtigated the probability of the theory on which ſuch practice was founded. A diſplacement of the abdominal viſcera, a diſeaſed ſtomach or ſpleen, might cauſe ſome degree of compreſſion; but when every part is in its natural ſituation, free from diſeaſe and enlargement, the compreſſion, if there is any, cannot prevent the circulation in the aorta, which is ſafely lodged in the angle made by the vertebræ and the ribs. Beſides, if ſuch a compreſſion really exiſted, the numerous branches which go off from the aorta deſcendens, while it is yet in the thorax, would be ſufficient to carry off the ſuperabundant blood, as the aorta is not repreſented in Dr. Fothergill's theory, or any defender of his opinions, to have the ſides compreſſed together. When Dr. Monro, in his Lectures, aſſerts that he can, by puſhing his hand againſt the abdomen, compreſs the aorta, and when the ſubclavian artery is totally compreſſed in amputation of the arm, why is not the patient, under theſe circumſtances, attacked with Apoplexy? If compreſſion by the diſtended ſtomach upon

* In Heiſter, we find the actual cautery, and ſcarification of the occiput, uſed as remedies for Apoplexy.

upon a great artery can produce that diſeaſe, why is not Apoplexy produced when the compreſſion is equally powerful upon the ſame, or an equally important, part of the arterial ſyſtem?

I am, &c.

*April* 10, 1802. JAMES ATKINSON.

